# Tobacco use among Latinx adolescents: exploring the immigrant paradox

**DOI:** 10.1186/s12887-018-1355-9

**Published:** 2018-11-30

**Authors:** Anna E. Epperson, Jan L. Wallander, Marc N. Elliott, Mark A. Schuster

**Affiliations:** 1 Stanford Prevention Research Center, Department of Medicine, Stanford University, 1265 Welch Road, Suite 300, Palo Alto, CA 94305 USA; 2 Psychological Sciences and Health Sciences Research Institute, University of California, Merced, 5200 Lake Rd, Merced, CA 95340 USA; 3 RAND Corporation, 1776 Main Street, Santa Monica, CA 90401 USA; 4 Division of General Pediatrics, Boston Children’s Hospital, 300 Longwood Ave, Boston, MA 02115 USA; 5 Department of Pediatrics, Harvard Medical School, 25 Shattuck St, Boston, MA 02115 USA; 6 Kaiser Permanente School of Medicine, 100 S. Los Robles Avenue, Pasadena, CA 91101 USA

**Keywords:** Latinxs, Immigrant paradox, Generational status, Tobacco use, Adolescent

## Abstract

**Background:**

Research suggests that an immigrant paradox exists where those who were not born in the United States (1^st^ generation) have significantly better health than those who were born in the U.S. (2^nd^ generation or more). The aim of the current study was to examine the immigrant paradox with respect to tobacco-related perceptions and parenting influences in smoking initiation among Latinx adolescents.

**Methods:**

Data came from the 7^th^ and 10^th^ grade Healthy Passages™ assessments of Latinx participants in three U.S. urban areas (*N* = 1536) who were first (18%), second (60%), and third (22%) generation. In addition to demographics, measures included perceived cigarette availability and peer smoking, intentions and willingness to smoke, and general monitoring by parents. Parents reported on generational status and their own tobacco use. The primary outcome was participant’s reported use of cigarettes.

**Results:**

By 10^th^ grade, 31% of Latinx youth had tried a cigarette, compared to 8% in 7^th^ grade. After controlling for age, gender, and socioeconomic status, regression analyses indicated that there were no significant differences related to generational status in cigarette smoking initiation in either 7^th^ or 10^th^ grade. Youth tobacco-related perceptions, general parental monitoring, and parental tobacco use predicted Latinx adolescent cigarette use initiation by 10^th^ grade.

**Conclusions:**

Latinx adolescents might not have deferential smoking rates based on generation status, suggesting that the immigrant paradox concept may not hold for smoking initiation among Latinx adolescents. Rather, factors influencing cigarette initiation generally in adolescents as a group appear to apply to Latinxs as well.

## Background

Over 37 million adults in the U.S. are current smokers [1] and most (80%) of these smokers began smoking cigarettes before the age of 17 [2]. Despite significant public health efforts to reduce the prevalence of smoking in the U.S., tobacco use continues to be the leading cause of preventable death and is associated with numerous negative health outcomes, including respiratory problems, lung cancer, and cardiovascular disease [3]. National health data suggest that time of smoking initiation varies by race/ethnicity [4], with Latinxs initiating much earlier than (non-Latinx) Whites. With thousands of youth beginning to smoke each day [2] and more male Latinx youth initiating cigarette smoking before age 13 years (13%) compared to their White (10%) and Black (10.5%) peers [4], there is a need to understand factors associated with smoking initiation during adolescence. Such information may help with the development of prevention efforts, especially those targeted at Latinx youth.

Previous research has indicated that immigrants to the U.S. and children of immigrants may be less likely to engage in behaviors that are harmful to health and may have a morbidity and mortality advantage compared to those without a recent immigration history, regardless of level of socioeconomic status or race/ethnicity [5]. This finding has been reported for a variety of health risk behaviors, including substance use and sexual risk behaviors [6–8], and health conditions [9, 10], such as mental health disorders and certain types of cancers and cancer outcomes. This phenomenon of engaging less frequently in risk behaviors and having better overall health in the current and recent immigrant generation is referred to as the *immigrant paradox* [11, 12]. Moreover, for adults, there is an overall mortality advantage, where 1^st^ and 2^nd^ generation Latinxs have a longer life expectancy compared to 3^rd^ generation Latinxs, defined as those born in the U.S. to parents born in the U.S. [13].

Despite studies documenting the immigrant paradox for a broad range of health issues, we are aware of only two studies that have examined the role of immigrant generational status on smoking among Latinx youth. Both studies used data from the National Longitudinal Study of Adolescent Health (Add Health), a national, longitudinal study following youth from 7^th^ grade into young adulthood. One study found evidence to support the paradox [14] after controlling for demographic covariates (age, gender, parental education, and household composition), whereas the other found no significant difference by generational status after controlling for other covariates, including parental control and parental smoking [15]. Consequently, conclusions regarding the existence of the paradox in relation to Latinx adolescent smoking are unclear. Findings for Latinx adults have also been mixed, with two studies reporting that 1^st^ [16, 17] and 2^nd^ [18] generation Latinxs were less likely to be smokers compared to 3^rd^ generation and that 3^rd^ generation have the highest overall tobacco use, but another study reporting no difference by generational status [19].

Previous research has shown that initiation of tobacco use among youth is associated with many sociodemographic factors and tobacco-related perceptions and attitudes. The theory of planned behavior is a widely used theoretical framework for understanding factors associated with smoking initiation among youth [20, 21] and links behavior with intentions and perceptions about external influences [22]. Adolescent tobacco use initiation has been shown to be strongly associated with availability of cigarettes [23], peer smoking [24, 25], parent smoking [26], parental monitoring [27], and intentions and willingness to smoke [28–30], with at least one previous study finding that intentions to smoke are associated with smoking among Latinx adolescents [30]. Further, having friends who smoke cigarettes [25] is associated with increased smoking intentions, willingness, and future initiation. Being closely monitored by a parent has also been found to be related to decreased smoking willingness and initiation [24]. Few studies, however, have examined the extent to which these influences are significant for Latinx adolescents specifically, and we know of none that considers immigrant generational status together with tobacco-related perceptions (intentions and willingness to smoke).

The current study updates previous work and examines tobacco use among Latinx adolescents beginning in middle school (7^th^ grade). We also examine the immigrant paradox together with demographic and parent and perceptual factors in cigarette smoking initiation. We hypothesized that among Latinx adolescents, (1) more recent immigrant generations (1^st^ vs. 3^rd^ and 2^nd^ vs. 3^rd^) evidence lower prevalence of smoking initiation by 7^th^ and 10^th^ grade and tobacco-related perceptions of willingness and intent to use in 7^th^ grade compared to 10^th^ grade. Based on past findings, we further hypothesized (2) that tobacco-related perceptions and parenting influences in 7^th^ grade predict, beyond generational status differences, smoking initiation by 10^th^ grade. Specifically, we examined as predictors perceptions of tobacco availability and peer smoking, future smoking intentions and willingness to smoke, and parental tobacco use and general monitoring.

## Methods

Data came from the Healthy Passages™ study, a longitudinal (2004–2011), multi-site cohort study of health and health behaviors in youth in 5^th^, 7^th^, and 10^th^ grades [31, 32].

### Participants

Fifth-grade students were recruited from public school classrooms in three locations (Birmingham, Alabama; Los Angeles, California; Houston, Texas) to participate in the Healthy Passages™ study. Using a two-stage probability sampling procedure, participants were selected. To ensure adequate sample sizes of students who identified as Black, Latinx, and White, schools within Birmingham, Los Angeles, and Houston were randomly selected with probabilities proportionate to a weighted measure of the scarcity of a school’s students relative to targets of these three racial/ethnic groups. Within these selected schools, all 5^th^ grade students were invited to participate [32]. Among the participants (and their parents/caregivers) that granted permission to be contacted and completed interviews in 5^th^ grade (*N* = 5147; 51% female; 35% Latinx), 4773 (93%) and 4521 (89%) completed follow-up assessments at the second (2 years later) and third (3 years later) wave, corresponding to when the participants generally were in 7^th^ and 10^th^ grade.

The analysis sample (*n* = 1536) contained participants who had reported no tobacco use at baseline (5^th^ grade), identified as Hispanic/Latinx based on their parent’s report, completed all three waves, and could be classified as first- (18.4%), second- (59.9%), or third (21.7%) generation (see below for definition). The sample (51% female) had a mean age of 11.13 (*SD* = 0.58) at 5^th^ grade, 13.10 (*SD* = 0.63) at 7^th^ grade, and 16.12 (*SD* = 0.64) at 10^th^ grade. Selected sample characteristics are shown in Table 1 (see [32] for further details).

**Table 1:** Sample characteristics (*N* = 1536)

	N	Wtd %
Female	774	50.5
Generational Status		
1^st^ Generation	272	18.4
2^nd^ Generation	883	59.9
3^rd^ Generation	381	21.7
Parental level of education		
Some HS or less	631	44.9
HS diploma/GED	360	23.6
Some college/2 year degree	351	21.8
4 year degree or higher	186	9.8
Parent household composition		
Single parent household	532	33.2
Two- parent household	1002	66.8

*Wtd* Weighted, % is calculated with weights to reflect sampling, *HS* High School, *GED* General Equivalence Diploma

### Procedure

Following standard procedures approved by the Institutional Review Boards at all study sites, two trained interviewers completed the Healthy Passages™ assessment protocol with the adolescent and one parent/caregiver at their home or another agreed upon location at each assessment. Written informed consent was provided by the parent, and the adolescent provided written assent. The interviews were conducted using both computer-assisted personal and self-interview procedures with the adolescent and parent separated in private spaces [31]. A Spanish version could be chosen by either at each assessment, except for youth at 10th grade (applied partly or fully at 5th grade: 8% of youth, 23% of parents; 7th grade: 4% of youth, 30% of parents; 10th grade: 30% of parents). Third-generation adolescent participants were the largest group to complete the interview mainly or entirely in English (98%), followed by second- (81%), and first- (50%) generation.

### Measures

The outcome of focus was **cigarette smoking initiation**, measured at 7^th^ and 10^th^ grade with the question, “Have you ever tried cigarette smoking, even one or two puffs?” (0 = *no;* 1 = *yes)*.

**Generational Status.** During enrollment in the study, each parent was asked whether they had been born inside or outside the U.S. Parents were also asked whether their child was born inside or outside the U.S. Based on a classification scheme described by Coll and Marks [11], the child was classified as one of the following: 1) *first-generation*, if both the participant (child) and the parent were born outside the U.S.; 2) *second-generation*, if the participant (child) was born in the U.S. but the parent was born outside the U.S.; and 3) *third-generation*, if both the participant (child) and the parent were born inside the U.S.

**Perceived peer smoking** was measured in 7^th^ grade with one question, “How many of your closest friends do you think have smoked cigarettes?” (1 = *none,* 3 = *many*). This was dichotomized into 0 = *no peer use* or 1 = *peer use*.

**Perceived cigarette availability** was assessed in 7^th^ grade with one question, “Has anyone ever offered you a cigarette?” (0 = *no* or 1 = *yes).*

**Intentions to smoke** were measured in 7^th^ grade with one question, “Do you think you will smoke cigarettes at any time during the next year?” with responses ranging from 0 = *no*, 1 = *mayb*e, or 2 = *yes.* This was recoded into a dichotomized variable with 0 = *no* and 1 = *maybe/yes*.

**Willingness to smoke** was measured in 7^th^ grade with one question, “If one of your closest friends offered you a cigarette, would you smoke it?” with responses ranging from 0 = *no*, 1 = *mayb*e, or 2 = *yes.* This was recoded into a dichotomized variable with 0 = *no* and 1 = *maybe/yes*.

**Monitoring** was measured in 7^th^ grade using five questions from a previous study [31] in which the adolescent was asked to indicate on a four-point scale (1 = *do not know much,* 4 = *know a lot*) how much his or her parent knew generally about what he or she did with free time (e.g., “How much do your parents know about where you are most afternoons after school?”) and who his or her friends were (e.g., “How much do your parents know about who your friends really are?”). The five items were summed with scores ranging from 5 to 20 (α = .80).

**Parent tobacco use** was measured in 7^th^ grade with two questions posed to the parent, “During the past 12 months, how many cigarettes did you smoke per day?” (*0 = none; 7 = more than 30 per day*) and, “During the past 12 months, did you use chewing tobacco, snuff, or dip, or smoke cigars or a pipe? (1 = *Yes*; 2 = *No*”). These were combined to create a dichotomized variable, where “None” (0) or “No*”* (2) on both questions was recoded as a “No” (0) and all other response combinations were coded as “Yes” (1).

**Control Variables.** Several covariates were included in the analysis including age, gender (male/female), highest level of education in household, and household composition. Highest level of education reported for either parent was classified into four categories ranging from less than high school graduation (1) to completion of a four-year college degree or higher (4). Household composition was coded based on parent report as either “two-parent” household or “other” (i.e., “single” parent).

### Data analysis

All analyses were conducted with design weights to account for differential probabilities of selection of students according to their school and a cluster variable to account for clustering of students within schools using IBM SPSS Statistics™ Complex Sampling module. Weighting also accounted for non-participation (by school, race/ethnicity, gender, and combinations thereof) initially, dropout, and differences between the retained sample (10^th^ grade) and the original sample (5^th^ grade), producing unbiased estimates among respondents if the characteristics used in the weights account for all nonresponse bias. Sensitivity analyses indicated that participants who completed all three waves (analytic sample) did not differ on any of the demographic or tobacco-related factors from participants who did not complete the second or third (final) wave except on highest level of education in household and household composition. For highest level of education in household, participants who did not complete all waves were more likely to have some high school education or less (49.7%) and not be from a two-parent household (48.3%) versus those who completed all waves (40.4 and 56.7%, respectively; *p’s* < .05). Two variables had missing data present [education (0.5%) and cigarette smoking initiation (8.1%)]; therefore, participants with missing data on these variables were excluded from the analysis. Analyses indicated they did not significantly differ from the analysis sample on any demographic variables. To further test the potential role of missing data, the multiple imputation method was used to estimate these missing values. This approach produced substantively identical results (available upon request).

Descriptive statistics and tests for group differences in tobacco use initiation, tobacco-related perceptions, and parental influences by generational status were conducted first using one-way ANOVA and chi-square tests. Logistic regression analyses followed to examine associations between tobacco-related perceptions and parental influences with tobacco use initiation in 7^th^ grade and to predict initiation in 10^th^ grade, starting with generational status (Model 1) and then adding in turn, tobacco-related perceptions (Model 2) and then parental tobacco use and general monitoring (Model 3). All models controlled for the covariates of age, gender, and household education and composition. Two-way interactions between generational status and other predictors (e.g., monitoring, perceived peer smoking) were tested in the model; interaction results are not presented as none were significant.

## Results

**Fig. 1:**
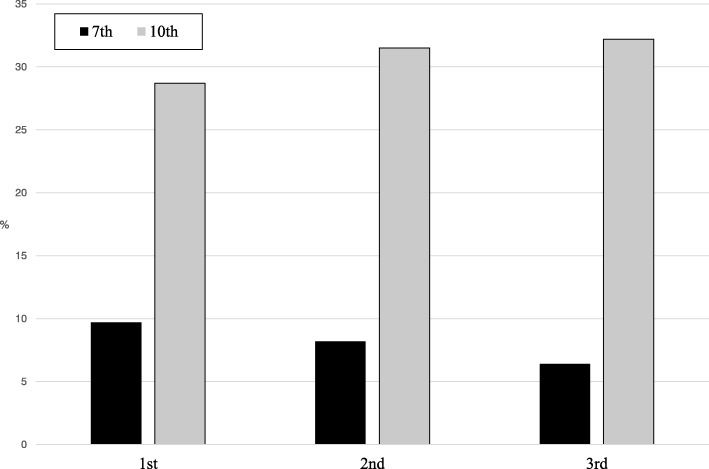
Prevalence of cigarette smoking initiation in 7th and 10th grade by immigration generation status

Descriptive statistics are presented in Table 1. As shown in Fig. 1, approximately 8% of the overall sample of Latinx adolescents reported having tried cigarette smoking by 7^th^ and 31% by 10^th^ grade. For smoking-related perceptions reported in the 7^th^ grade, 34% reported that they believed their friends smoked cigarettes, 16% reported having been offered a cigarette, 9% reported that they had future intentions to smoke and 8% that they would be willing to smoke in the future if offered cigarettes (Table 2). Seventeen percent of parents reported using tobacco.

**Table 2:** Tobacco-related variables by generational status (*N* = 1536)

	Overall		1^st^ Generation	2^nd^ Generation	3^rd^ Generation	
	N	Wtd.%	Wtd.%	Wtd. %	Wtd. %	χ^2^
Total	1536	–	18.4	59.9	21.7	
7^th^ grade Cigarette Smoking Initiation	118	8.1	9.7	8.2	6.4	2.34
10^th^ grade Cigarette Smoking Initiation	430	31.2	28.7	31.5	32.2	0.92
Perceived Cigarette Availability	243	16.1	18.1	15.5	16.3	1.14
Perceived Peer Smoking	513	33.8	28.9	34.1	37.4	4.95
Intentions to Smoke	134	8.7	7.7	9.1	8.4	0.58
Willingness to Smoke	123	8.4	7.4	8.3	9.5	0.93
Parent Tobacco Use	256	16.8	13.9^a^	15.8^b^	22.2^c^	**9.15**
	*M (SE)*					*F*
Monitoring	16.81 (0.11)		16.87(0.14)^a,b^	16.68(0.13)^a^	17.12(0.17)^b^	**2.38**

*Wtd* Weighted, % is calculated with weights to reflect sampling, *HS* High School

^a,b,c^Different superscripts within generational status subgroups for row variable indicates statistically significant difference as per *χ2* or *F* tests. Boldface indicates statistical significance (*p* < .05)

### Generational status

Results (Tables 2 & 3) indicated that there were no significant differences in cigarette smoking initiation among Latinx youth of varying generational status in 7^th^ (χ^2^ [2, *N* = 1536] = 2.34, *p* = .39) or 10^th^ grade (χ^2^ [2, *N* = 1536] = 0.92, *p* = .73). There were no significant differences among generational status groups in tobacco-related perceptions or general parental monitoring or tobacco use (*p*’s > .05).

**Table 3:** Logistic Regression Model for Tobacco Use Initiation by 7th and 10th Grade

	Cigarette Smoking					
	7^th^ Grade			10^th^ Grade		
OR (95% CI)	Model 1	Model 2	Model 3	Model 1	Model 2	Model 3
*Generational Status*						
1^st^ generation	Ref.	Ref.	Ref.	Ref.	Ref.	Ref.
2^nd^ generation	0.93 (0.48, 1.78)	0.73 (0.32, 1.64)	0.69 (0.30, 1.57)	1.29 (0.85, 1.95)	1.18 (0.77, 1.81)	1.15 (0.75, 1.77)
3^rd^ generation	0.70 (0.36, 1.34)	0.48 (0.21, 1.07)	0.46 (0.20, 1.03)	1.26 (0.77, 2.07)	1.21 (0.72, 2.04)	1.17 (0.70, 1.97)
Age	**1.80 (1.25, 2.59)**	1.12 (0.81, 1.55)	1.11 (0.79, 1.55)	1.61 (1.37, 1.90)	**1.29 (1.09, 1.52)**	**1.28 (1.08, 1.52)**
Female (referent: male)	0.79 (0.51, 1.24)	0.71 (0.44, 1.13)	0.78 (0.49, 1.24)	0.98 (0.74, 1.31)	0.98 (0.70, 1.36)	1.03 (0.74, 1.45)
*Parental level of education*						
Some HS or less	Ref.	Ref.	Ref.	Ref.	Ref.	Ref.
HS diploma/GED	1.49 (0.72, 3.07)	1.21 (0.55, 2.68)	1.15 (0.52, 2.55)	1.30 (0.84, 2.02)	1.25 (0.80, 1.94)	1.24 (0.79, 1.93)
Some college	1.27 (0.59, 2.71)	0.92 (0.36, 2.36)	0.91 (0.36, 2.35)	1.29 (0.84, 1.98)	1.15 (0.74, 1.81)	1.12 (0.70, 1.78)
4 year degree or higher	1.63 (0.73, 3.64)	1.37 (0.55, 3.41)	1.23 (0.49, 3.04)	1.39 (0.95, 2.02)	1.26 (0.86, 1.87)	1.18 (0.79, 1.76)
Single parent household (referent: two-parent)	**1.66 (1.13, 2.42)**	1.18 (0.75, 1.85)	1.18 (0.75, 1.85)	**1.30 (1.01, 1.68)**	1.12 (.85, 1.49)	1.08 (0.82, 1.42)
*Perceptions /Attitudes*						
Cigarette availability	**2.36 (1.34, 4.13)**		**2.21 (1.25, 3.90)**		**2.00 (1.40, 2.87)**	**1.91 (1.33, 2.74)**
Peer smoking	**3.05 (1.88, 4.94)**		**3.08 (1.89, 5.04)**		**1.74 (1.26, 2.40)**	**1.72 (1.25, 2.37)**
Future smoking intentions	**3.72 (2.23, 6.21)**		**3.62 (2.18, 6.00)**		**2.62 (1.70, 4.04)**	**2.53 (1.67, 3.83)**
Smoking willingness	**4.25 (2.64, 6.83)**		**3.54 (2.14, 5.86)**		**2.25 (1.33, 3.80)**	**1.92 (1.16, 3.18)**
*Family Influences*						
Monitoring			**0.90 (0.84, 0.96)**			**0.92 (0.88, 0.96)**
Parental Tobacco Use			1.44 (0.81, 2.55)			**1.59 (1.08, 2.34)**

All models controlled for: gender, child age in years at 7^th^ grade, parent household composition, and parent education. *OR* odds ratio, *CI* confidence interval. Model 1: Generation status and covariates only; Model 2: Tobacco use attitudes and perceptions added; Model 3: Parental influences added

Boldface indicates statistical significance (*p* < .05)

### Adolescent tobacco-related perceptions

As shown in Table 3, Model 2 for cigarette smoking, Latinx adolescents who reported cigarette availability in the 7^th^ grade were at least twice as likely to report initiating cigarette smoking in both the 7^th^ and 10^th^ grades compared to those who did not report being offered cigarettes (*p* = .004, *p* = .001, respectively). Believing that friends smoked cigarettes was associated with a higher likelihood of having initiated cigarette smoking by 7^th^ grade (*p* < .001), and was predictive of cigarette smoking initiation by 10^th^ grade (*p* = .001). Both intentions to smoke in the next year and willingness to smoke if offered cigarettes were associated with a higher likelihood of initiating smoking by 7^th^ grade (*p*’s < .05), and Latinx adolescents reporting intentions and willingness were twice as likely to smoke by 10^th^ grade (*p* < .001 and *p* = .012, respectively).

### Parental influences

As shown in Table 3, Model 3, when added to the regressions predicting tobacco use in 7^th^ and 10^th^ grade, having increased general parental monitoring was associated with a decreased odds of cigarette smoking initiation in 7^th^ grade (*p* = .001) and predicted a lower likelihood of trying cigarettes in the 10^th^ grade (*p* < .001). Finally, parental tobacco use was predictive of cigarette smoking in the 10th grade (*p* = .021), but was not associated with cigarette smoking in the 7^th^ grade (*p* = .208). Perceptual variables generally remained significant predictors of tobacco use when parental variables were added to the regression model.

### Demographic influences

Increased age was associated with an increased odds of cigarette smoking initiation in 10^th^ grade (*p* = .005) but was not associated with cigarette smoking in the 7^th^ grade (*p* = .544). Gender, level of education, and household composition were not significantly associated with cigarette smoking in the 7^th^ or 10^th^ grades (*p*’s > .05).

## Discussion

Cigarette smoking among Latinx adolescents increased almost four-fold between 7th (ages 12–13) and 10th (15–16) grade, from 8 to 31%. However, there is little evidence for the immigrant paradox accounting for this pattern, at least when based on the adolescents’ generational status. Smoking in Latinx youth in 10th grade was predicted, as expected, by tobacco-related cognitive processes present in 7th grade pertaining to peer norms, cigarette availability, and intentions and willingness to try cigarettes, as well as by a parent who uses tobacco and provides less general monitoring.

Our finding that there were not significant differences between Latinx adolescents of varying generational status for smoking initiation is consistent with one previous study that controlled for demographic, behavioral, and parental factors [15], but is in contrast with the second study that found that 3^rd^ generation adolescents were more likely to smoke cigarettes compared to 2^nd^ and 1^st^ generation [14]. Our findings may have differed as our study was conducted in three specific metropolitan areas in the U.S, while previous work used national data from Add Health. Although not previously examined, we also found that there were no differences in tobacco-related perceptions (intentions and willingness) due to generational status. The association between these tobacco-related cognitive processes reported in middle school (7^th^ grade) and smoking initiation in high school (10^th^ grade) supports previous findings that youth who report intentions and willingness to smoke in the future are more likely to initiate smoking [27, 28]. Finally, results indicating that perceptions about cigarette availability, peer and parental smoking, and parental general monitoring predicted smoking initiation also supported previous findings among Black, Latinx, and White adolescents [29, 30].

Several limitations in this research should be noted. The longitudinal cohort design hinders causal inferences based on these findings. As noted previously, this study was conducted in three specific metropolitan areas in the U.S, and caution should be exercised in generalizing to other populations. Further, Latinx youth in this study predominantly have Mexican and Central America heritage, also raising caution about generalizing to Latinx groups with other origins. Immigration status was only recorded for one parent. Further, generational status is often used as a proxy for acculturation which may have limitations. Acculturation is a complex psychological and sociological process that has multiple dimensions and may be better assessed with both psychometric measures and specific age of migration for youth and both parents. Tobacco use and all covariates, except generational status, were measured by youth self-report.

Despite these limitations, this is one of the first studies to examine longitudinally how generational status measured in middle school is associated with tobacco use in high school. It is the first study we know of to examine generational status together with sociodemographic and tobacco-related perceptions and attitudes. The current study differs from previously published work by examining both smoking-related future intentions and willingness to smoke and actual initiation of smoking among Latinx adolescents from primary school (5^th^ grade) to middle school (7^th^ grade). Our results add to previous findings suggesting that the immigrant paradox may not apply specifically for cigarette smoking among Latinx youth. Although findings from this study do not support the concept of the immigrant paradox, results suggest that believing that cigarettes were available and having family or friends who smoked increased the likelihood that Latinxs would try cigarette smoking by 10^th^ grade. This has implications for smoking prevention efforts with this population, where interventions should aim to address these social and perceptual influences. The association with perceived cigarette availability may be due to increased access to tobacco for school age youth, as previous research has shown that tobacco retailers are often clustered in higher concentrations near schools [33]. Strong policies banning the sale of tobacco and enforcement of these policies for neighborhoods with schools and large school-aged populations could protect youth from tobacco products.

## Conclusion

Finding no significant differences in tobacco initiation and related perceptions due to generational status and finding that predictors of tobacco initiation for Latinx youth are highly similar to those demonstrated repeatedly in general samples of youth suggest that there is little basis for substantially different prevention approaches. Therefore, current tobacco use prevention efforts mainly implemented through public schools are likely to be applicable to Latinx youth with varying personal and family migration histories. Nonetheless, there may be benefits to certain cultural adaptations of generally applicable prevention programs, such as those used for HIV-prevention and alcohol abuse programs among Latinxs [34, 35]. Our findings raise questions about the immigrant paradox as it applies to tobacco use for Latinx adolescents. It is hoped that these findings and this line of research may help practitioners and researchers further determine effective components of prevention and intervention efforts.

## Abbreviations

Add Health: National Longitudinal Study of Adolescent Health
HIV: Human immunodeficiency virus
